# Supplementary material for: Development of bitopic nanobody-ligand conjugates targeting G protein-coupled receptors and exhibiting logic-gated signaling
Source: PLoS Biol. 2025 Nov 20;23(11):e3003424. doi: 10.1371/journal.pbio.3003424 (PMC12633870; doi:10.1371/journal.pbio.3003424)
Supplement: Attachment — Submitted filename: Responses to reviewers A2AR 08132025.docx [file pbio.3003424.s006.docx]

REVIEWS:

Reviewer #1 (Bryan L Roth, signs review): This is an imaginative and interesting paper that provides a new technology to create modified GPCRs which can function, perhaps, as logic gates. I enjoyed reading this paper and pondering its implications which I think are major.

I have only a couple of technical issues which do not distract from my overall enthusiasm for this work, but which are essential to understand further the potential mechanism(s) involved.

Response: We thank Dr. Roth for his careful review, encouraging comments, and helpful technical questions below.

DETAILED AND RELATIVELY MINOR TECHNICAL COMMENTS:

1. FIG 2: did the Nb conjugate alter cell surface receptor expression?

Response: One important point regarding internalization is that unlike most GPCRs, A2AR has been reported to be deficient for beta-arrestin recruitment and to internalize very weakly. Thus we focused on the internalization of PTHR1 by bivalent Nb-ligand conjugates. We have added new experiments testing whether application of Nb-conjugates causes changes in levels of cell surface PTHR1 (Supplementary Figure 11). These data show only PTH1-34 (a full agonist, previously reported to induce receptor internalization) induces internalization of PTHR1. In contrast, none of the Nb-ligand conjugates tested induce disappearance of cell surface PTHR1. Whether Nb-ligand conjugates are defective at inducing GPCR internalization when targeting other receptor pairs remains to be tested and represents an interesting area for future investigation. Text describing these new data and interpretation have been added with a quotation below:

“Although past work had shown that Nb-ligand conjugates in which both Nb and ligand targeted PTHR1 were unable to induce receptor internalization, it was unclear whether Nb-ligand conjugates co-targeting A2AR and PTHR1 might behave differently. To probe this question, we performed a cell-based enzyme-linked immunosorbent assay (ELISA) to measure the disappearance of receptor from the cell surface upon treatment with agonists (Supplementary Figure 11). Only the prototypical PTHR1 agonist PTH1-34 stimulated a decrease in the level of cell surface PTHR1. In contrast, neither PTH1-11-NbALFA nor CGS-NbPTHR1 reduced the level of PTHR1 detected at the cell surface. This observation may be connected to the previous finding that PTH1-11-Nb conjugates failed to induce β-arrestin recruitment [ref]. It is also relevant that A2AR activation does not stimulate substantial recruitment of β-arrestin or receptor internalization [refs]. Previous work demonstrating that bivalent conjugates targeting CXCR7 (a GPCR) can induce target internalization [ref] suggest that further studies will be beneficial in revealing contexts in which bivalent conjugates stimulate target downregulation.”

2. The kinetics of activation and inactivation (Figs 2D,F; 3, 4) shoud be replicated with a direct measurement of receptor-G protein coupling (e.g. BRET sensor) to clarify the mechanism (e.g. direct effect on receptor-effector coupling).

Response: We agree that a kinetic assessment that reports directly on G protein activation (rather on a downstream second messenger) offers a useful viewpoint on GPCR signaling. We have now added kinetic data on Gαs dissociation for dose-response data shown in Supplementary Figure 5. Our efforts to establish an assay system to report on Gαs activation, to follow both ligand induced Gs activation and the persistence of G protein activation after ligand washout, were unsuccessful. Further technical optimization will be necessary on our end and is the subject of ongoing work.

We have added new text describing these data and a discussion of possible future directions, quoted below:

“Ligand-induced changes in BRET signals persisted for the full length of the assay (30 m), indicating enduring stimulation of G protein activation (Supplementary Figure 5c-e). Future studies will benefit from extension of BRET biosensor assays such as TRUPATH to report on the dynamics of G protein activation following ligand washout [refs].”

3. Also need to assess effects of conjugation on cell surface receptor expression levels for all experiments as it is not entirely clear that the data in Supplementary Fig 6 provides this data for all constructs.

Response: We have added a new figure showing the relative level of expression of each receptor construct through staining with a fluorescently labeled analogue of CGS21680 (see Supplementary Figure 4). Cell lines expressing different variants of A2AR are stained at levels above baseline with a fluorophore-CGS21680 conjugate synthesized for this analysis. Some variants of A2AR with N-terminal appendages (epitope tags, nanobodies) express at levels higher than wild type A2AR while GFP-A2AR expresses at a reduced level relative to wild type A2AR. It is not clear whether these differences result from properties inherent to the presence of the tags at the N-terminus of constructs or if this variation is solely a result of the clonal cell selection process, which can stochastically give rise to clonal cell lines with different levels of receptor expression even when expressing identical receptors. We have added new text referring to these data, quoted below:

“Each of these cell lines exhibited staining with a fluorophore conjugated analogue of CGS21680 (CGS-AZ647), with A2AR-BC2-6E-ALFA, or A2AR-Nb6E-ALFA showing the highest levels of staining (Supplementary Figure 4).”

Reviewer #2: In this manuscript, Sachdev et al. describe the development of bitopic ligands for the adenosine A2A receptor (A2AR) by using a nanobody against a genetically encoded epitope tag on the extracellular domain of the receptor. The A2A receptor is part of the G protein coupled receptor (GPCR) family which is of broad therapeutic interest. In this work, the authors expand on their prior research, extending the strategy of nanobody-peptide conjugation to a small molecule ligand (CGS21680). This agonist is attached to nanobodies targeting various epitope tags or the extracellular domain of the PTH receptor. In each case, the signaling properties of CGS are altered by its nanobody tethering, in particular showing prolonged activation. Finally, the authors show that using a nanobody targeting one receptor linked to CGS (or a dynorphin peptide) allows "logic-gated" signaling, in which activation of one receptor is contingent on co-expression of another.

Overall, the manuscript is clearly written and interesting. The topic of antibody-drug conjugates is timely and will likely be of interest to a broad readership including researchers in GPCR pharmacology and drug development. The concept of logic-gated activation is clearly demonstrated with several distinct examples provided. While generally thorough, a notable gap in the work is a relatively limited investigation of arrestin activation and signaling bias generally. The authors have shown previously that Nb-peptide conjugates can exhibit strong and unexpected biased signaling, so it would be helpful to see some investigation of this. Specifically, the long duration of action for A2aR-targeted CGS conjugates could in principle reflect differences in arrestin recruitment in addition to direct effects of ligand dissociation kinetics.

Response: We thank this reviewer for their careful evaluation and positive perspective. We have responded to specific points below.

Major points:

An investigation (or at a minimum discussion) of biased signaling possibilities for the A2a-targeted conjugates would be appropriate. Some arrestin recruitment assays are provided in the section on using alternative receptors for targeting, but this could be expanded particularly in the context of prolonged receptor activation.

Response: We fully agree with the reviewer that investigations of nanobody-ligand conjugate biased signaling responses is of substantial interest. In this work we chose to focus on biased agonist responses for mu opioid receptor instead of A2A-receptor because A2AR has been shown to signal predominantly through a single pathway (Gαs signaling) with minimal/undetectable signaling through other pathways (including negligible β-arrestin recruitment) (see ref. Avet et al eLife 2022). We have added text (quoted below) to clarify our choices and emphasize past findings that guided these choices.

“Past work with Nb ligand conjugates targeting PTHR1 revealed highly selective activation of cAMP signaling without concomitant induction of β-arrestin recruitment (biased agonism) [ref]. Past work has shown that unlike most GPCRs, activation of A2AR does not stimulate arrestin recruitment [ref]. Hence, β-arrestin recruitment assays were not performed for conjugates targeting this pair of receptors.”

Did the authors examine effects on receptor trafficking when dual-specificity agonists are used? For example, does activation of A2a receptor by CGS force internalization of GLP1R or PTHR? This could be quite important if so, and others have shown that GPCRs can force internalization of other cell surface proteins (Pance et al., Nature Biotech 2022).

We thank the reviewer for raising this important point and sharing the useful reference. Past work with nanobody-ligand conjugates targeting PTHR1 (Sachdev *et* al Nat Comms 2024) showed that such bitopic ligands did not induce beta-arrestin recruitment or stimulate receptor internalization. To address this question for nanobody-ligand conjugates that engage two distinct targets (described for the first time in this manuscript) we performed new experiments. These new data (Supplementary Figure 11) show that Nb-PTH conjugates that target A2AR and PTHR1 (NbAlfa-PTH1-11) fail to stimulate internalization of PTHR1, whereas it effectively stimulated cAMP responses. Further studies in future work will be necessary to see whether conjugates targeting other GPCR pairs can effectively stimulate receptor internalization. We have added new text to describe these findings (with a new citation) as quoted below:

“Although past work had shown that Nb-ligand conjugates in which both Nb and ligand targeted PTHR1 were unable to induce receptor internalization, it was unclear whether Nb-ligand conjugates co-targeting A2AR and PTHR1 might behave differently. To probe this question, we performed a cell-based enzyme-linked immunosorbent assay (ELISA) to measure the disappearance of receptor from the cell surface upon treatment with agonists (Supplementary Figure 11). Only the prototypical PTHR1 agonist PTH1-34 stimulated a decrease in the level of cell surface PTHR1. In contrast, neither PTH1-11-NbALFA nor CGS-NbPTHR1 reduced the level of PTHR1 detected at the cell surface. This observation may be connected to the previous finding that PTH1-11-Nb conjugates failed to induce β-arrestin recruitment [ref]. It is also relevant that A2AR activation does not stimulate substantial recruitment of β-arrestin or receptor internalization [refs]. Previous work demonstrating that bivalent conjugates targeting CXCR7 (a GPCR) can induce target internalization [ref] suggest that further studies will be beneficial in revealing contexts in which bivalent conjugates stimulate target downregulation.”

The authors describe varying the linker length when developing ligands with agonism of the PTH1R and A2AR, but generally do not comment on initial linker length development of their CGS-Nb. Is this something that did not need to be optimized in this case? If so, it may be worth mentioning.

We chose a linker length that was similar to the linker used in optimized peptide-CGS conjugates used to target A2AR in past work (see Cabalteja *et al* Bioconj Chem 2022). The data presented in Supplementary Figure 13 (formerly Supplementary Figure 10) suggests that linker length is not an important determinant of bitopic conjugate signaling activity, at least in the context of these constructs. Additional work in future studies will be required to see if linker length is an important variable for activity in other bitopic conjugates targeting GPCRs. We have added new text to emphasize this precedent for linker length and the need for further studies in the future.

“Whether the composition and length of the linker connecting Nb to ligands in heteromeric conjugates was important for biological activity was also tested. Our initial choice of linker length was based on previous studies from our group using CGS-peptide conjugates, in which a medium-length PEG linker was effective [ref].”

Minor points:

This paper focuses on presenting functional signaling data of the bitopic ligands developed, which is very common in papers describing antibody drug conjugates but some biochemical characterization of the nanobody ligand conjugate could be interesting to see how biochemical affinities are affected in addition to signaling potency. This would also be important if the authors chose to investigate quantitative analysis of signaling bias.

Response: We have now added new data comparing the affinity of a Nb (Nb6E) and the Nb6E-CGS conjugate for immobilized 6E epitope. Nb6E-CGS and Nb6E bind to their targets in an ELISA with nearly identical affinities (Supporting Figure 3). These data suggest that the appendage of a ligand (CGS) to a nanobody has a minimal impact on epitope binding. We have added new text describing these findings, quoted below.

“An assessment of CGS-Nb_6E_ for binding to its epitope (6E tag) using an enzyme-linked immunosorbent assay (ELISA) revealed that it retained high affinity target binding, with affinity very similar to Nb_6E_ (Supplementary Figure 3). This observation suggests that the style of ligand linkage used in this study does not negatively impact the affinity of Nbs for their epitopes.”

The click chemistry method to producing nanobody ligand conjugates is of great interest and will be quite useful in future development of bitopic ligands, it would be interesting if the authors stated the percent labeling efficiency of the method.

Response: We agree that a thorough characterization of the synthetic procedures, reaction variables, and percentage yields for conjugate formation would be useful for the research community. We respectfully suggest that a comprehensive assessment is beyond the scope of this work. A single test reaction performed to synthesize Nb6E-biotin-CGS resulted in a recovered yield of 40%. This information has been added to the Supporting Methods section.

“Nb-DBCO conjugates (produced using sortagging, described above) were mixed with 1.5 equivalents of CGS-PEG3-azide in PBS buffer. The reaction proceeded for 2-3 h at which time the product (CGS-Nb conjugate) was purified using a Cytiva PD-10 SephadexTM G 25M size exclusion column to remove residual CGS-PEG3-azide. The CGS-Nb conjugates were characterized by mass spectrometry (see Supplementary Table 2). The typical yield for this type of azide-DBCO click reaction was 40%.”

The structure diagrams in Fig 2 are a bit confusing. The surface representation of the receptor makes it difficult to see that the molecules shown are specifically tethered to the receptor.

Response: We acknowledge that these receptor constructs are confusing. We have attempted to improve clarity by modifying this graphic. We have added arrows to show the point at which N-terminal tags engrafted upon the wild type A2AR receptor in Figure 2.
